# Chlorahololide D, a Lindenane-Type Sesquiterpenoid Dimer from *Chloranthus holostegius* Suppressing Breast Cancer Progression

**DOI:** 10.3390/molecules28207070

**Published:** 2023-10-13

**Authors:** Ying Li, Wenhui Liu, Jing Xu, Yuanqiang Guo

**Affiliations:** 1 State Key Laboratory of Medicinal Chemical Biology, College of Pharmacy, and Tianjin Key Laboratory of Molecular Drug Research, Nankai University, Tianjin 300350, China; liying@baridd.ac.cn (Y.L.); 2120201241@mail.nankai.edu.cn (W.L.); 2 State Key Laboratory of Functions and Applications of Medicinal Plants, Guizhou Medical University, Guiyang 550014, China

**Keywords:** *Chloranthus holostegius*, lindenane-type sesquiterpenoid dimer, breast cancer, FAK, zebrafish model

## Abstract

Aimed at discovering small molecules as anticancer drugs or lead compounds from plants, a lindenane-type sesquiterpene dimer, chlorahololide D, was isolated from *Chloranthus holostegius*. The literature review showed that there were few reports on the antitumor effects and mechanisms of chlorahololide D. Our biological assay suggested that chlorahololide D blocked the growth and triggered apoptosis of MCF-7 cells by stimulating the reactive oxygen species (ROS) levels and arresting the cell cycle at the G2 stage. Further mechanism exploration suggested that chlorahololide D regulated apoptosis-related proteins Bcl-2 and Bax. Moreover, chlorahololide D inhibited cell migration by regulating the FAK signaling pathway. In the zebrafish xenograft model, chlorahololide D was observed to suppress tumor proliferation and migration significantly. Considering the crucial function of angiogenesis in tumor development, the anti-angiogenesis of chlorahololide D was also investigated. All of the research preliminarily revealed that chlorahololide D could become an anti-breast cancer drug.

## 1. Introduction

Up to the present, breast cancer has officially replaced lung cancer as the most ubiquitous cancer among women in the world [1]. According to the latest statistics in the “IARC Biennial Report 2020–2021” published by International Agency for Research on Cancer (IARC) [2], the proportions of new cases and deaths of breast cancer in global cancers had reached 24.5% and 15.5% up to 2020, ranking the first and fifth places, respectively [3,4]. Breast cancer has seriously threatened the health and life of women around the world. However, owing to the rapid proliferation and metastasis, the cure rate of breast cancer is very low. At present, chemotherapy, radiotherapy, and surgery are still the main treatment methods for breast cancer. Among them, the universal application of chemotherapy, as the most widespread method, still faces challenges due to the recurrence, tolerance, and severe side effects. Early diagnosis and treatment are the key to reducing the mortality of breast cancer. Novel drugs with lower toxicities and higher efficiencies are urgently needed to prevent and treat breast cancer.

Traditional Chinese medicines have been widely recognized for their uses in cancer treatment [5,6]. Significant progress has been made in the use of structurally rich chemical components in traditional Chinese medicine for clinical anticancer therapy. A tremendous amount of work has gone into discovering antineoplastic natural products. Inspired by the clinical treatment of natural cancer medicines like podophyllotoxin and vinblastine, we are committed to discovering natural lead compounds with anticancer activity from medicinal plants.

*Chloranthus holostegius* (Hand.-Mazz.) Pei et Shan, belonging to the genus *Chloranthus* (Chloranthaceae), is a folk Chinese herbal medicine mainly used for treating bone bruises and injuries, paralysis, rubella, and liver wind headache [7]. With the development of modern pharmacology, it has been discovered that *C. holostegius* contains rich bioactive components with diverse structures [7,8]. It is well attested that lindenane-type sesquiterpenoids and derivatives are the characteristic chemical constituents of *C. holostegius* [8,9,10]. Among them, lindenane-type sesquiterpenoid dimers with different skeletons are biosynthesized through different biological pathways from two lindenane-type monomers, and the most usual step is involving in an endo-Diels–Alder cycloaddition [11,12]. Lindenane-type sesquiterpenoid dimers with the skeleton from intramolecular [4 + 2] cycloadditions are the most common [7,8], which usually exhibit significant biological activities in anticancer, anti-inflammation [13], K^+^ current inhibition [14], anti-HIV [15], anti-malaria [16], and liver protection [17]. By virtue of the complex and novel skeletons, aside from natural products, a total synthesis of lindenane [4 + 2] dimers with an intramolecular six-membered ring has also been reported [18,19]. During our discovery of novel natural products, several [4 + 2] type lindenane-type sesquiterpenoid dimers were purified from the herbal plant of *C. holostegius*, which were identified as chlorahololide D [10], sarcandrolide A [20], and shizukaol E [20], and an MTT screening against two tumor cells (HepG2 and MCF-7) was carried out. It was found that chlorahololide D showed the most inhibitory activity toward MCF-7 cells. Furthermore, based on the *in vitro* screening, in-depth research on the antitumor activity of chlorahololide D was conducted using a zebrafish model, attempting to elucidate its antitumor mechanism.

## 2. Results

**Figure 1:**
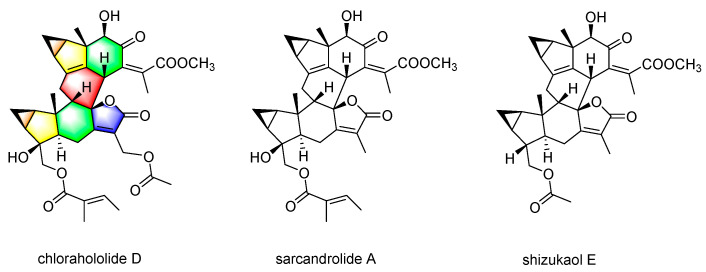
Chemical structure of chlorahololide D, sarcandrolide A, and shizukaol E.

Three lindenane-type sesquiterpene dimers, chlorahololide D [10], sarcandrolide A [20], and shizukaol E, were isolated from *C. holostegius*. The structures are elucidated as shown in Figure 1. The ^1^H and ^13^C NMR spectra are given in the Supplementary Material.

### 2.1. Chlorahololide D Inhibited Cancer Cell Growth In Vitro

The *in vitro* biological activity of chlorahololide D against human tumor cell lines was preliminarily evaluated by an MTT method. Etoposide was employed as the positive control in this assay. The 50% inhibiting concentration (IC_50_) listed in Table 1 demonstrated that only chlorahololide D possessed strong *in vitro* activity as expected (IC_50_ no more than 20 μM) with IC_50_ values of 13.7 and 6.7 μM for HepG2 and MCF-7 cells, respectively, while sarcandrolide A and shizukaol E, two lindenane-type sesquiterpenoid dimers from *C. holostegius*, showed weaker activities than chlorahololide D (Figure 1). According to the MTT assay, chlorahololide D with the lowest IC_50_ for MCF-7 cell line (6.7 μM) was adopted for the subsequent experiments.

### 2.2. Chlorahololide D Induced Apoptosis of MCF-7 Cells

**Figure 2:**
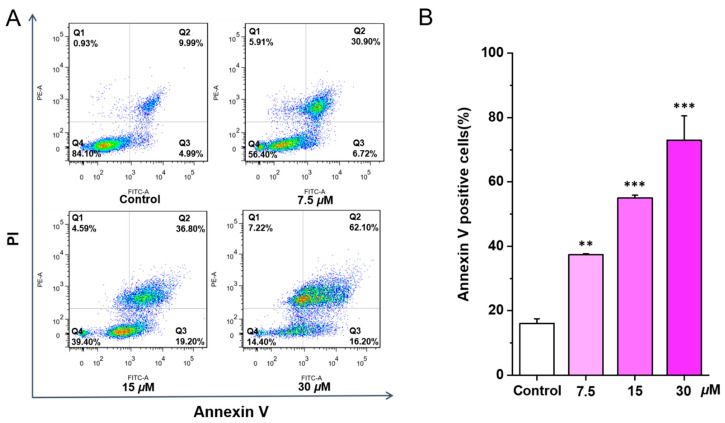
Chlorahololide D triggered apoptosis in MCF-7 cells. Various concentrations of chlorahololide D (7.5, 15, and 30 μM) were administrated to MCF-7 cells and incubated for 48 h. Cells were stained with Annexin V and propidium iodide (PI), and detected by flow cytometry subsequently. (**A**) Flow cytometric analysis of MCF-7 cells with the treatment of chlorahololide D. (**B**) Histogram of the proportions of apoptotic cells at 48 h after being treated with chlorahololide D. The results are expressed as mean ± SD. ** *p* < 0.01 and *** *p* < 0.001 vs. control group.

After confirming the cytotoxicity, flow cytometry was further employed to detect whether the cytotoxicity of chlorahololide D on MCF-7 cells was due to apoptosis induction. As depicted in Figure 2, the total number of apoptosis cells stagnated in Q2 and Q3 regions raised intuitively with the increasing treatment concentrations of chlorahololide D (7.5, 15, and 30 μM). The quantification analysis via ImageJ software (ImageJ 1.51k) revealed that the percentages of apoptosis cells increased in a dose-dependent pattern from 16.0% (control group) to 37.4% (7.5 μM), 55.1% (15 μM), and 73.0% (30 μM), respectively, indicating that chlorahololide D could trigger apoptosis of MCF-7 cells.

### 2.3. Chlorahololide D Increased Cellular ROS

**Figure 3:**
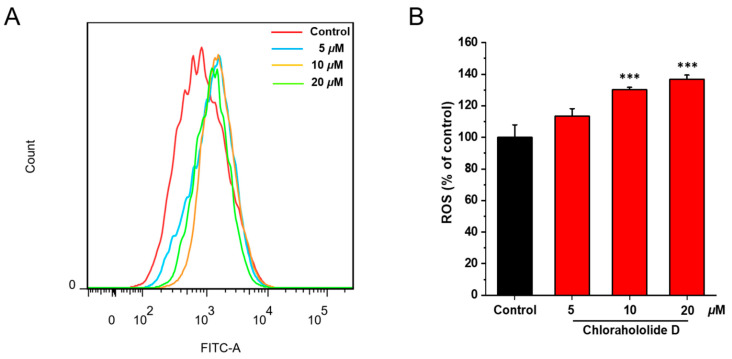
Chlorahololide D increased ROS production in MCF-7 cells. MCF-7 cells were incubated with chlorahololide D (5, 10, and 20 μM) for 48 h. MCF-7 cells were stained with DCFH-DA and detected by the flow cytometer. (**A**) Flow cytometric analysis of MCF-7 cells after being treated with chlorahololide D. (**B**) Histogram of relative ROS levels. The results are expressed as mean ± SD. *** *p* < 0.001 vs. control group.

Apoptosis of tumor cells is generally accompanied by the increasing production of ROS. To explore whether chlorahololide D promoting the apoptosis of MCF-7 cells was in relation to ROS levels, flow cytometry was performed. DCFH-DA was used as a probe. As illustrated in Figure 3, after being administrated with chlorahololide D for 24 h, ROS levels were 1.13 (5 μM), 1.30 (10 μM), and 1.36 (20 μM) times that of the blank control group, respectively. 

### 2.4. Chlorahololide D Arrested MCF-7 Cell Cycle

**Figure 4:**
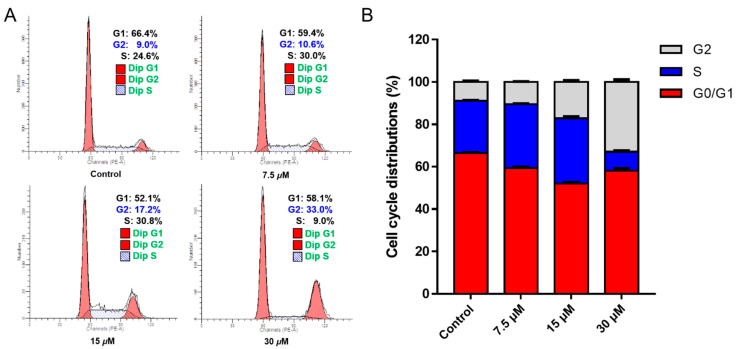
Chlorahololide D arrested G2 phase in MCF-7 cells. MCF-7 cells were treated with chlorahololide D (7.5, 15, and 30 μM) for 48 h. (**A**) The cells were harvested and stained with propidium iodide (PI), and the cell cycle distribution was detected using flow cytometry. (**B**) Histogram of cell cycle phases distribution. Data from three separate experiments are presented as mean ± SD.

Flow cytometry was carried out to examine whether chlorahololide D induced cell apoptosis by arresting the cell cycle. From Figure 4A, it can be clearly observed that MCF-7 cells were arrested in the G2 stage upon treatment of various concentrations of chlorahololide D (7.5, 15, and 30 μM). In addition, data analysis based on the histogram (Figure 4B) showed that the proportions of cells in the G2 phase were significantly increased from 9.0% (control group) to 10.6% (7.5 μM), 17.2% (15 μM), and 33.0% (30 μM), respectively. These results suggested that the cell cycle was arrested at the G2 stage by chlorahololide D to cause the apoptosis of MCF-7 cells.

### 2.5. Chlorahololide D Affected the Expression of Apoptosis-Related Proteins

**Figure 5:**
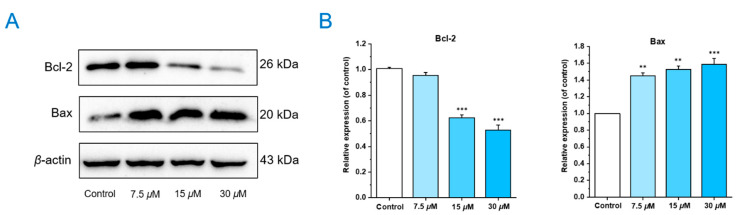
Chlorahololide D regulated apoptosis-related proteins. MCF-7 cells were pre-treated with chlorahololide D for 36 h, and Western blotting analysis was carried out. (**A**) The expression of Bcl-2 and Bax. (**B**) Histogram of the protein relative expression levels. *β*-actin protein was used as an internal reference. The results are presented as mean ± SD. ** *p* < 0.01 and *** *p* < 0.001 vs. control group.

The effects of chlorahololide D on the expression of anti-apoptotic Bcl-2 protein and pro-apoptotic Bax protein were examined via Western blotting. The results shown in Figure 5 were as expected. After being treated with chlorahololide D, the protein levels of Bcl-2 decreased while the expression levels of Bax increased in MCF-7 cells. It was deduced that chlorahololide D prompted apoptosis by regulating the apoptosis-related proteins Bcl-2 and Bax in MCF-7 cells. 

### 2.6. Chlorahololide D Inhibited MCF-7 Cell Metastasis by Regulating FAK Signaling Pathway

**Figure 6:**
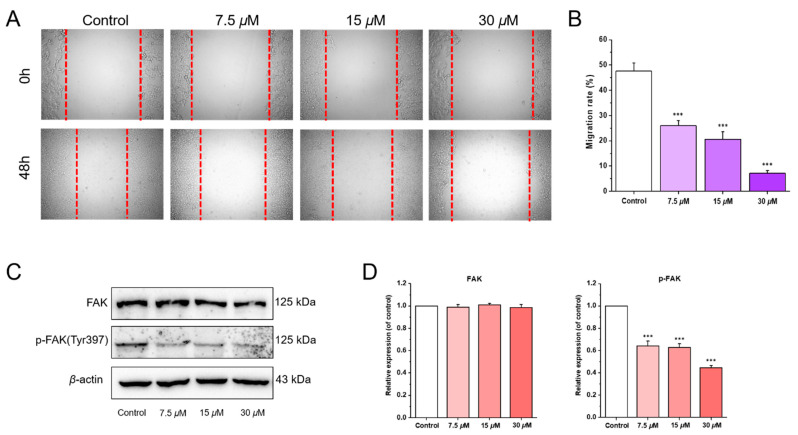
Chlorahololide D inhibited MCF-7 cells migration via regulating migration-related proteins. MCF-7 cells were pre-treated with chlorahololide D for 48 h, and Western blotting analysis was carried out. (**A**) MCF-7 cells were photographed at 0 h and 48 h. (**B**) Histogram of the migration rate (%). (**C**) The expression of FAK and p-FAK. (**D**) Histogram of the protein relative expression levels. *β*-actin protein was an internal reference. The results are expressed as mean ± SD.*** *p* < 0.001 vs. control group.

Except for inhibiting proliferation and inducing apoptosis, antineoplastic drugs are usually involved in the mechanism of inhibiting metastasis of tumors. A wound-healing assay was performed in this study to detect the inhibitory effects of chlorahololide D on cell migration. As shown in Figure 6A, after incubation with chlorahololide D for 48 h, the migration of MCF-7 cells was inhibited to varying degrees. The outcome, after being deeply analyzed with ImageJ software (Figure 6B) exhibited that the migration rates were reduced by 1.83, 2.31, and 6.68 times that of the control group from 7.5 to 15 and 30 μM. 

A Western blotting experiment was employed to further explore the influence of chlorahololide D on migration-related proteins. As shown in Figure 6C,D, with the increasing concentrations of chlorahololide D, no significant impact was observed on the expression of FAK protein, but a dose-dependent reduction occurred in the p-FAK levels.

### 2.7. In Vivo Antitumor Activity of Chlorahololide D Using a Zebrafish Model

**Figure 7:**
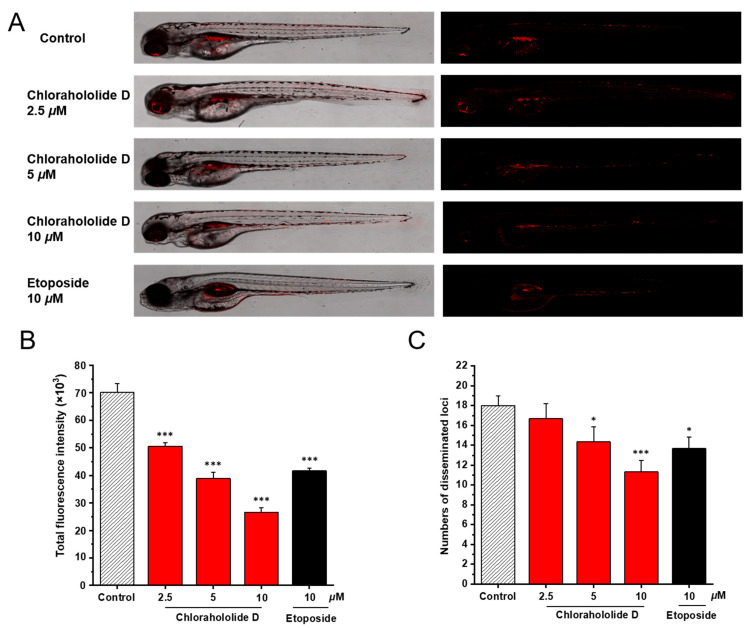
Chlorahololide D inhibited proliferation and migration *in vivo*. CM-DiI stained MCF-7 cells were microinjected into 2 dpf zebrafish embryos. After 4 h, tumor-bearing embryos were treated with chlorahololide D (2.5, 5, and 10 μM) for 48 h (*n* = 15/group). Etoposide (10 μM) was used as the positive control. (**A**) Intensity and distribution of the red fluorescence were photographed using a confocal microscope of disseminated foci in zebrafish. (**B**) The proliferation was quantified by ImageJ software. (**C**) The metastasis of MCF-7 cells was analyzed using ImageJ software. All of the results are expressed as the mean ± SD. * *p* < 0.05 and *** *p* < 0.001 vs. the control group.

The similarity of signal pathways related to tumors between humans and zebrafish allows the zebrafish xenograft model to be ideal for examining the antitumor activity of chlorahololide D *in vivo*. MCF-7 cells stained with CM-DiI were administrated into the yolk sac of 2 dpf embryos, and the yolk sac of the zebrafish embryo grew under normal conditions, revealing the zebrafish xenograft model was developed successfully. Etoposide served as the positive control here. After treatment with chlorahololide D (2.5, 5, and 10 μM), the intensity and distribution of the red fluorescence were both inhibited obviously (Figure 7A). Further, data from Figure 7B,C showed that the relative intensity and the foci of red fluorescence were reduced from 72.0% to 38.0% and from 92.6% to 62.9%, respectively, when compared with the blank control group. Notably, at the same concentration (10 μM), chlorahololide D had stronger inhibitory effects than etoposide. The results indicated that chlorahololide D suppressed tumor proliferation, invasion, and metastasis.

### 2.8. Antiangiogenetic Activity of Chlorahololide D Using a Transgenic Zebrafish Model

**Figure 8:**
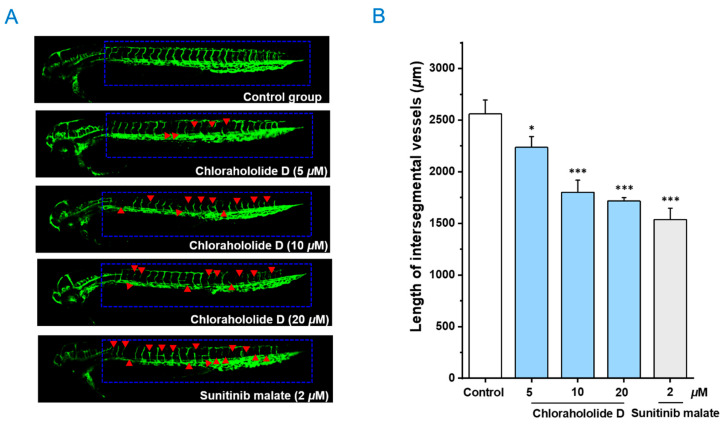
The embryos of transgenic zebrafish *Tg*(*fli1: EGFP*) were treated with the positive control, sunitinib malate (2 μM), and chlorahololide D (5, 10, and 20 μM) for 48 h. (**A**) Representative images of ISVs in zebrafish under a confocal microscope. (**B**) The average length of ISVs in zebrafish after treatment with sunitinib malate and chlorahololide D (5, 10, and 20 μM) (*n* = 15/group). All of the results are expressed as the mean ± SD. * *p* < 0.05 and *** *p* < 0.001 vs. the control group.

Considering the important role of angiogenetics in the process of tumor growth, the effects of chlorahololide D on intersegmental vessel formation in transgenic zebrafish *Tg(fli1: EGFP)* were estimated. As revealed in Figure 8, treatment with different doses of chlorahololide D (5, 10, and 20 μM) caused varying degrees of destruction on the intersegmental vessels (ISVs) and dorsal longitudinal anastomotic vessels (DLAVs). It could be observed that both ISVs and DLAVs were perceptibly absent and broken in Figure 8A (pointed out with red arrows). The statistical outcome via ImageJ software in Figure 8B revealed the average length of ISVs under chlorahololide D were 2559.8 ± 135.3 μm (0 μM), 2235.4 ± 106.0 μm (5 μM), 1801.1 ± 116.6 μm (10 μM), and 1715.9 ± 32.3 μm (20 μM), respectively, which displayed a concentration-dependent reduction. In summary, chlorahololide D blocked the neoplasm proliferation, invasion, and metastasis by inhibiting angiogenesis.

## 3. Materials and Methods

### 3.1. Biological Materials and Cell Culture

Fetal bovine serum (FBS, BI, Israel) and Dulbecco’s modified Eagle’s medium (DMEM) were provided by LABBIOTECH Co., Ltd. (Shandong, China). Dimethyl sulfoxide (DMSO) and 3-(4,5-Dimethylthiazol-2-yl)-2,5-diphenyltetrazolium bromide (MTT) were purchased from Solarbio (Beijing, China). Cell tracker CM-DiI was obtained from Yeasen Biotechnology Co., Ltd. (Shanghai, China). Annexin V-FITC apoptosis detection kit, cell cycle and apoptosis kit, and BCA protein assay kit were supplied by Beyotime Biotechnology Co., Ltd. (Shanghai, China). Rabbit monoclonal antibodies against FAK, p-FAK (Tyr 397), Bax, Bcl-2, and *β*-actin were purchased from Cell Signaling Technology (Danvers, MA, USA). HepG2 and MCF-7 were acquired from Shanghai Institutes for Biological Sciences, Chinese Academy of Sciences (Shanghai, China). The cells were cultured in DMEM containing 10% (*v*/*v*) FBS and 100 U/mL penicillin/streptomycin under a water-saturated atmosphere of 95% air and 5% CO_2_. Adult AB and transgenic zebrafish were obtained from Shanghai Feixi Biotechnology Co., Ltd. (Shanghai, China).

### 3.2. Extraction, Isolation, and Purification

The details of chemical materials as well as extraction, isolation, and purification of the plants of *C. holostegius* are supplied in the Supplementary Materials.

Chlorahololide D: ^1^H NMR (400 MHz, CDCl_3_): *δ*_H_ 2.05 (1H, m, H-1), 1.00 (1H, m, H-2*α*), 0.30 (1H, dd, *J* = 7.2, 4.0 Hz, H-2*β*), 1.83 (1H, m, H-3), 3.92 (1H, d, *J* = 3.1 Hz, H-6), 3.95 (1H, s, H-9), 1.88 (3H, s, H_3_-13), 1.02 (3H, s, H_3_-14), 2.78 (1H, d, *J* = 6.2 Hz, H-15*α*), 2.59 (1H, m, H-15*β*), 1.57 (1H, m, H-1′), 0.71 (1H, ddd, *J* = 14.5, 8.7, 5.9 Hz, H-2′*α*), 1.27 (1H, m, H-2′*β*), 1.50 (1H, m, H-3′), 1.77 (1H, dd, *J* = 13.7, 5.9 Hz, H-5′), 2.35 (1H, dd, *J* = 18.6, 5.9 Hz, H-6′*α*), 2.73 (1H, dd, *J* = 18.6, 13.7 Hz, H-6′*β*), 1.86 (1H, m, H-9′), 4.82 (1H, d, *J* = 13.0 Hz, H-13′a), 4.77 (1H, d, *J* = 13.0 Hz, H-13′b), 0.86 (3H, s, H_3_-14′), 4.21 (1H, d, *J* = 11.6 Hz, H-15′a), 3.81 (1H, d, *J* = 11.6 Hz, H-15′b), 6.90 (1H, m, H-3″), 1.85 (1H, d, overlapped, H-4″), 1.86 (3H, s, H_3_-5″), 3.76 (3H, s, H_3_-OMe), 2.08 (3H, s, H_3_-COMe); ^13^C NMR (400 Hz, CDCl_3_): *δ*_C_ 25.7 (C-1), 15.9 (C-2), 24.7 (C-3), 142.3 (C-4), 131.8 (C-5), 40.9 (C-6), 147.6 (C-7), 200.4 (C-8), 80.2 (C-9), 51.1 (C-10), 131.5 (C-11), 170.4 (C-12), 20.3 (C-13), 15.2 (C-14), 25.3 (C-15), 25.3 (C-1′), 11.9 (C-2′), 28.3 (C-3′), 77.5 (C-4′), 60.4 (C-5′), 22.6 (C-6′), 172.1 (C-7′), 93.2 (C-8′), 55.7 (C-9′), 44.7 (C-10′), 123.6 (C-11′), 171.3 (C-12′), 55.0 (C-13′), 26.3 (C-14′), 70.7 (C-15′), 168.3 (C-1″), 128.0 (C-2″), 138.8 (C-3″), 14.5 (C-4″), 12.1 (C-5″), 52.5 (C-OCH_3_), 170.3 (C-OOCCH_3_), 20.4 (C-OOCCH_3_).

### 3.3. Cytotoxic Activity Assay

The MTT assay was designed to evaluate the cytotoxic activity. The details of this experiment are described in the Supplementary Materials.

### 3.4. Apoptosis Analysis by Flow Cytometry

Flow cytometry was employed to analyze the effects of chlorahololide D on the MCF-7 cell’s apoptosis. The details were supplied in the Supplementary Materials.

### 3.5. Measurement of Reactive Oxygen Species (ROS)

The intracellular ROS levels were detected by the ROS assay kit (Beyotime, Shanghai, China) using a flow cytometry experiment. The details of this experiment are described in the Supplementary Materials.

### 3.6. Cell Cycle Analysis

The distribution of the cell cycle of MCF-7 cells affected by chlorahololide D was performed by flow cytometric analysis. The details are provided in the Supplementary Materials.

### 3.7. Western Blotting Analysis

Western blotting was used to determine the levels of protein expression including Bax, Bcl-2, FAK, and p-FAK. The experimental procedures for Western blotting analysis are supplied in the Supplementary Materials.

### 3.8. Wound-Scratch Assay

Wound-scratch assay was routinely performed for the detection of the migration ability of MCF-7 cells. The experimental process is stated in the Supplementary Materials.

### 3.9. In Vivo Anti-Tumor Assay

The anti-tumor activity of chlorahololide D on the tumor cell proliferation and metastasis *in vivo* was evaluated by the zebrafish xenograft model based on the reported method. The particular processes are provided in the Supplementary Materials.

### 3.10. Antiangiogenetic Assay Using Transgenic Zebrafish Model

The angiogenesis inhibitory effects of chlorahololide D were conducted in transgenic zebrafish *Tg* (*fli1: EGFP*) embryos. All operating steps of this experiment are described in the Supplementary Materials.

### 3.11. Statistical Analysis

GraphPad Prism is use to analyze data. All the data presented as mean ± SD. Probabilities *p* < 0.05 indicated to be significant by analysis of variance (ANOVA). One-way ANOVA multiple comparisons were used to analyze the differences between three or more groups. There were three repetitions of each experiment.

## 4. Discussion

In the present study, three natural products possessing the same skeleton of lindenane-type sesquiterpenoid dimer were isolated from *C. holostegius*. An MTT assay revealed that chlorahololide D exhibited potent cytotoxicity against HepG2 and MCF-7 cell lines, and had more cytotoxic effects against MCF-7 cells with an IC_50_ value of 6.7 μM. Chlorahololide D possessed stronger activity than most of those natural antitumor products we have reported, like xipsxanthone H against A549, HepG2, K562, and HeLa cells (IC_50_, 15.3, 16.1,15.0, and 17.3 μM) [21], cratoxylumxanthone C against A549, HepG2, and MCF-7 cells (IC_50_, 17.5, 17.7, and 21.9 μM) [22], kurzipenes D against HepG2 and K562 cells (IC_50_, 9.7 μM and 7.2 μM), and xipsxanthone D against HeLa cells (IC_50_, 9.6 μM) [23]. Subsequently, a series of experiments were designed and carried out to assess the effects of chlorahololide D on MCF-7 cells *in vitro* and *in vivo*. Chlorahololide D could promote apoptosis and block the proliferation and migration of MCF-7 cells *in vitro*. Furthermore, an experiment based on the zebrafish model, a recognized and credible preclinical animal model, demonstrated that chlorahololide D also inhibited the proliferation and metastasis of MCF-7 cells *in vivo*. The results above indicated that it was worthwhile to explore the antitumor mechanism of chlorahololide D deeply.

Inducing apoptosis is an important mechanism of antineoplastic effects of chemotherapy drugs [24]. ROS and cell cycle are considered to be closely related to cell apoptosis [22,25]. ROS including peroxides, superoxides, hydroxyl radicals, singlet oxygen, and *α*-oxygen, are small molecules with high activities and a short lifespan in cells [26]. When produced excessively in cells, ROS will bring damage to proteins, nucleic acids, lipids, cell membranes, and organelles in cells, thereby inducing apoptosis [27]. In addition, an imbalance of the cell cycle usually causes cell apoptosis [25]. Different physical and chemical stimuli have different degrees of damage to cells in different cycles, and cells in various cycles also have different repair abilities to resist damage, both of which result in cell cycle specificity [28]. Some antitumor drugs cause cell apoptosis precisely by specifically blocking the cell cycle [29]. For example, trigothysoid N and cratoxylumxanthone C were both found to arrest the A549 cell cycle at the G0/G1 stage, while xipsxanthone H blocked the cell cycle at the S stage in the same cell line [21,22,30]. Kurzipene D blocked the HepG2 cell cycle at the S stage [31], and curcin C made U2OS cells stagnate both in the S and G2/M phases [32]. Our study showed that chlorahololide D could stimulate ROS production and arrest the MCF-7 cell cycle in the G2 phase, which may be an explanation for chlorahololide D inducing apoptosis.

Bcl-2 family in the mitochondrial pathway plays a significant role in apoptosis. Bcl-2 and Bax, a pair of the most important regulatory genes with opposing functions in the mitochondria-mediated apoptosis pathway in the Bcl-2 family, are responsible for apoptosis promotion and suppression [33], respectively. Our results showed that chlorahololide D significantly prompted apoptosis in MCF-7 cells. Apart from this, the outcome of Western blotting pointed out that chlorahololide D could decrease the production level of the Bcl-2 protein and increase the Bax protein level dose-dependently, suggesting the promotional effects of chlorahololide D on apoptosis may be related to the downregulation of the Bcl-2 level and upregulation of the Bax level in the mitochondrial pathway.

In addition to proliferation, invasion and metastasis are another two fatal difficulties in the cure of malignant tumors. Also known as protein tyrosine kinase 2 (PTK2), focal adhesion kinase (FAK) plays a vital role in tumor invasion and metastasis. Stimulation with growth factors or integrins leads to self-phosphorylation on the Y397 point at the N-terminus of FAK protein (Tyr397), which results in the activation of FAK [34]. Subsequently, the downstream signaling pathway is regulated in a kinase-dependent manner or the focal-adhesion-associated protein (FAAP) is recruited in a kinase-independent manner, thereby regulating tumor growth, invasion, and metastasis [35,36]. Therefore, FAK is considered as an important target for the development of anti-metastatic agents. Results showed that chlorahololide D significantly inhibited the migration by inhibiting the phosphorylation of FAK.

The importance of angiogenesis in the occurrence and development of tumor tissues is widely recognized. Inhibiting angiogenesis in tumors can limit the oxygen and nutrients required for tumor growth [37]. Based on this point, many antitumor drugs are developed to exert their effects. After demonstrating the *in vivo* antitumor effects of chlorahololide D using a zebrafish xenograft model, further analysis was conducted on its anti-angiogenesis effects in a transgenic zebrafish model. Chlorahololide D was turned out to significantly suppress the formation of blood vessels, thereby exerting antitumor effects.

The current study investigated the antitumor effects of chlorahololide D and revealed that this compound had strong cytotoxic activity on both MCF-7 and HepG2 cells (IC_50_ values, 6.7 and 13.7 μM). According to the literature, chlorahololide D was reported to have moderate cytotoxic activity toward the HeLa cell line (IC_50_ = 32.2 μM) [38]. In summary, chlorahololide D showed inhibitory effects on various tumor cells, which had different sensitivities to different tumor cells. The subsequent *in vivo* experiments confirmed the antitumor activity, and a series of investigations on the antitumor mechanism of chlorahololide D conducted may provide a reference and basis for future research of chlorahololide D as an antitumor agent.

## 5. Conclusions

With the increasing threat to human life and health from breast cancer and the shortage of ideal therapeutic drugs, it is urgent to develop drugs for breast cancer. Researchers place hopes on natural products, the natural resource pool for drug development. Chlorahololide D is a lindenane-type sesquiterpenoid dimer isolated from *C. holostegius*, which has significant cytotoxicity on MCF-7 cells. Furthermore, by increasing ROS level and blocking the cell cycle at the G2 phase, chlorahololide D significantly induced cell apoptosis. The mechanistic examination showed that chlorahololide D regulated the expression of apoptosis-related proteins (Bax and Bcl-2) to induce apoptosis. In addition, chlorahololide D inhibited cell migration by regulating the FAK signaling pathway. In addition, the *in vivo* antitumor effects and mechanism of chlorahololide D were confirmed using zebrafish xenograft and transgenic zebrafish models. Although the activity evaluation and preliminary mechanism study of chlorahololide D have been investigated in this paper, deeper exploration needs to be sought to promote its antitumor application in breast cancer.

## Figures and Tables

**Table 1:** Cytotoxicities of three compounds against two human cancer cell lines.

Compound	IC_50_ (μM)	
	HepG2	MCF-7
Chlorahololide D	13.7 ± 1.4	6.7 ± 1.0
Sarcandrolide A	40.6 ± 1.2	23.0 ± 3.3
Shizukaol E	34.8 ± 4.4	>60
Etoposide ^a^	15.7 ± 1.6	33.3 ± 5.0

^a^ Positive control. All results are presented as mean ± SD.

## Data Availability

Data will be made available on request.

## Supplementary Materials

The following supporting information can be downloaded at: https://www.mdpi.com/article/10.3390/molecules28207070/s1, Figure S1. 1H NMR spectrum of chlorahololide D; Figure S2. ^13^C NMR spectrum of chlorahololide D; Figure S3. ^1^H NMR spectrum of sarcandrolide A; Figure S4. ^13^C NMR spectrum of sarcandrolide A; Figure S5. ^1^H NMR spectrum of shizukaol E; Figure S6. ^13^C NMR spectrum of shizukaol E.

## Sample Availability

Not available.

## Abbreviations

ROS: reactive oxygen species; IARC, International Agency for Research on Cancer; FBS, Fetal bovine serum; ANOVA, analysis of variance; ISVs, intersegmental vessels; DLAVs, dorsal longitudinal anastomotic vessels.
