# Supplementary material for: Chlorahololide D, a Lindenane-Type Sesquiterpenoid Dimer from Chloranthus holostegius Suppressing Breast Cancer Progression
Source: Molecules. 2023 Oct 13;28(20):7070. doi: 10.3390/molecules28207070 (PMC10608938; doi:10.3390/molecules28207070)

# Supplementary material for

# Chloraholide D, a Lindenane-Type Sesquiterpenoid Dimer from *Chloranthus holostegius* Suppressing Breast Cancer Progression

## Contents


1. Extraction, isolation, and purification
2. Chemical materials
3. Cytotoxic activity assay
4. Apoptosis analysis by flow cytometry
5. Measurement of ROS
6. Cell cycle analysis
7. Western blotting analysis
8. Wound-scratch assay
9. *In vivo* anti-tumor assay using zebrafish model
10. Antiangiogenetic assay using transgenic zebrafish model
11. Statistical analysis
12. Spectra of chlorahololide D, sarcandrolide A, and shizukaol E

## 1. Extraction, isolation, and purification

The plants of *C. holostegius* were collected from Guangxi province, China, in October 2021 and authenticated by Y.G. (College of Pharmacy, Nankai University). A voucher specimen (No. 20211009CH) was deposited at the Laboratory of Natural Medicines, Nankai University.

The dried whole plants of *C. holostegius* (3.0 kg) were refluxed under 98% methanol (MeOH) (3×20 L). After solvent removal under reduced pressure, a crude extract (240 g) was afforded. The crude extract was suspended in $H_2O$ (360 mL) and partitioned with ethyl acetate (EtOAc). After removing the solvent, the total organic layer (102.5 g) was subjected to column chromatography over silica gel, and a gradient solvent system of petroleum ether (PE)-acetone was used for elution. Nine fractions ($F_1$–$F_9$) were attained based on the TLC analysis. Fraction $F_7$ (5.3 g) was separated by medium pressure liquid chromatography (MPLC) over octadecylsilane (ODS) (63–93% MeOH in $H_2O$) to give nine subfractions $F_{7\text{-}1}$–$F_{7\text{-}9}$. $F_{7\text{-}5}$ was further purified by preparative HPLC (equipped with a YMC-pack ODS-AM column, 20 × 250 mm, 78% MeOH in $H_2O$) to yield chlorahololide D ($t_R$ = 34 min, 12.6 mg), sarcandrolide A ($t_R$ = 37 min, 20.2 mg), and shizukaol E ($t_R$ = 40 min, 52.7 mg).

## 2. Chemical materials

Silica gel (200-300 mesh) was obtained from Qingdao Haiyang Chemical Group Co., Ltd. (Qingdao, People's Republic of China). Medium pressure liquid chromatography (MPLC) was run on a P0100 pump with an ultraviolet (UV) detector (Huideyi Co., Beijing, People's Republic of China) and a column (40 × 400 mm) filled by octadecylsilyl (ODS, 50 $\mu$m, YMC Co., Ltd.). High performance liquid chromatography (HPLC) was conducted on a CXTH system, provided with a Shodex RI-102 detector (Showa Denko Co., Ltd., Tokyo, Japan) and a YMC-pack ODS-AM (20 × 250 mm) column (YMC Co. Ltd., Kyoto, Japan). 1D NMR experiments were conducted on a Bruker AV 400 equipment (Bruker, Switzerland, 400 MHz for $^1$H and 100 MHz for $^{13}$C) with TMS as an internal reference at 25 °C. All of the chemical reagents (analytical grade) mentioned in the experiments were purchased from Tianjin Chemical Reagent Co. (Tianjin, People's Republic of China) and Sigma Co., respectively.

### 3. Cytotoxic activity assay

The cytotoxic activities were evaluated using an MTT assay. Briefly, after reaching approximate 80% confluence, the cells were harvested and seeded in 96-well plates ($1\times10^5$ cells/well) and allowed to adhere for 24 h at 37 °C. Then, the cells were treated with the test samples dissolved in DMSO at different concentrations, including the positive. Etoposide was used as a positive control. After continuous incubation for 48 h, 20 $\mu$L MTT solution (5 mg/mL) was added to each well for 4 h incubation. Then, the medium was replaced with 150 $\mu$L DMSO, and the absorbance was measured at 492 nm using a microplate reader (Thermo Fisher Scientific Inc. America). The experiments were performed in triplicate, and the $IC_{50}$ value was defined as the concentration of the compounds that inhibited cell proliferation by 50%.

### 4. Apoptosis analysis by flow cytometry

The apoptosis analysis of MCF-7 cells induced by the tested compound was accomplished by flow cytometry using Annexin V-FITC Apoptosis Detection Kit (Beyotime, C1062L) according to the manufacturer's instructions. Briefly, MCF-7 cells were harvested and seeded in 12-well plates ($1 \times 10^5$ cells/well) and allowed to adhere for 24 h at 37 °C. Then, the cells were treated with various concentrations (7.5, 15, and 30 $\mu$M) of chloraholide D. After 48 h incubation, the cells were washed twice with PBS and resuspended in the binding buffer (Beyotime, Shanghai, China). This suspension was incubated for 20 min at room temperature in the dark after adding 5 $\mu$L Annexin V-FITC and 10 $\mu$L PI. Then, cell apoptosis was examined by BD LSRFortessa flow cytometry (BD Biosciences). The cell apoptosis data were obtained with FLOWJO flow cytometry analysis software (FLOWJO LLC, Ashland, OR, USA).

### 5. Measurement of ROS

The realease of intracellular ROS was measured by flow cytometer. Briefly, MCF-7 cells were inoculated in 12-well plates ($1 \times 10^5$ cells per well) and cultured under appropriate conditions (5% $CO_2$, 37 °C) for 24 h. Then, MCF-7 cells were treated with chloraholide D (5, 10, and 20 $\mu$M) for 48 h. Then, the cells were collected, washed with PBS, and stained with 10 $\mu$M DCFH-DA probe (Beyotime, Shanghai, China) for 20 min at 37 °C. Finally, the cells were washed three times with serum-free culture medium and subjected to the flow cytometer (BD Biosciences). The ROS release was analyzed according to the FlowJo flow cytometry analysis.

### 6. Cell cycle analysis

The distribution of the cell cycle of MCF-7 cells affected by chloraholide D was performed using flow cytometric analysis. MCF-7 cells in the exponential growth phase were seeded in a 12-well plate

at the density of 2 × $10^5$ cells/well for 24 h at 37 °C. Then, the cells were treated with different concentrations of chloraholide D (7.5, 15, and 30 $\mu$M). After exposure to the test sample for 48 h, the cells were harvested, washed with PBS twice, and fixed in 70% ice-cold ethanol at 4°C overnight. Then, the cells were washed with PBS twice and treated with propidium iodide staining buffer containing RNase (Beyotime, C1052) for 30 minutes at 37°C in the dark, followed immediately by cellar DNA analysis using BDLSR Fortessa flow cytometry. Data were processed using ModFit LT Software.

**7. Western blotting analysis**

MCF-7 cells were seeded in a 6-well plate at the density of 2 × $10^5$ cells/well for 24 h. Then, the cells were treated with chloraholide D for 36 h, and the cells were washed with cold PBS twice and collected. The cells were lysed with lysis buffer containing protease inhibitor cocktail and PMSF. Then, the lysates were centrifuged at 12,000 rpm for 10 min, and the supernatants were collected to acquire the total protein. Protein concentrations were quantified using the BCA protein assay kit (Beyotime, P0012S). The proteins were separated by 10% SDS-PAGE and transferred to polyvinylidene difluoride. The membrane was blocked with 5% skim milk for 1 h at room temperature and then incubated (4 °C, overnight) with primary antibodies against Bax (Cell Signaling Technology, 14796S), Bcl-2 (Cell Signaling Technology, 4223S), FAK (Cell Signaling Technology, 3285S), and phospho-FAK (Tyr 397) (Cell Signaling Technology, 8556S). The membranes were subsequently incubated with horseradish peroxidase-conjugated secondary antibody for 1 h at room temperature. Lastly, the protein blots were visualized using an ECL detection kit (Beyotime, P0018AS). $\beta$-actin protein (Cell Signaling Technology, 4970S) was used as an internal reference. Each band was quantified by ImageJ software.

**8. Wound-scratch assay**

The effects of chloraholide D on motility ability of MCF-7 cells were assessed using wound scratch assay. Briefly, cells were seeded into a 6-well plate with a concentration of 5 × $10^5$ cells/well. After reaching 90% confluence, cells were scratched by a sterile pipette tip. Then, the cells were treated with various concentrations of chloraholide D. The wounded healing was observed using microscope at 0 and 48 h. The scratch distance value was measured by ImageJ software.

**9. *In vivo* anti-tumor assay using zebrafish model**

Embryos were obtained from adult AB zebrafish as reported previously. 48 hours post-fertilization (hpf) embryos were utilized to establish a xenograft tumor model. Prior to microinjection, MCF-7 cells were labeled with 2 mM CM-DiI (Yeasen, 40718ES50). Then, the cells were resuspended in serum-free DMEM medium and adjusted to a density of 1 × $10^7$ cells/mL. Subsequently, 48 hpf embryos were

anesthetized and microinjected into the yolk with 5 nL stained cells. After 4 h incubation, tumor-bearing embryos were randomly divided into five groups (15/group) and treated with different concentrations of chloraholide D by soaking. The embryos were incubated continuously for 48 h at 28.5 °C. Lastly, images were captured at 5 day post-fertilization using confocal microscopy (Leica, Germany), and the density and focus number of red fluorescence were measured using ImageJ software.

**10. Antiangiogenetic assay using transgenic zebrafish model**

The angiogenesis inhibitory activity of the selected compound was carried out using transgenic zebrafish *Tg* (*fli1: EGFP*). Transgenic zebrafish were obtained from Shanghai FishBio Co., Ltd. The embryos were obtained from adult *Tg* zebrafish as reported previously. Briefly, 6 hpf embryos were grouped randomly and placed in the 12-well plate, and then the embryos were exposed to various concentrations of chloraholide D for 48 h at 28.5 °C. After the treatment, embryos were anesthetized with 0.02% tricaine and photographed by a confocal microscope (Leica, Germany). The development of intersegmental blood vessels (ISVs) and dorsal longitudinal anastomotic vessels (DLAVs) at 48 hpf were observed, and the length of ISV vessels was measured using Image J software.

**11. Statistical analysis**

Data were analyzed by GraphPad Prism and presented as mean ± SD. Probabilities (P) less than 0.05 were determined to be significant by analysis of variance (ANOVA). The differences among three or more groups were analyzed by one-way ANOVA multiple comparisons. The experiments were repeated three times.

## 12. Spectra of chloraholide D

### Figure S1. $^{1}H$ NMR spectrum of chloraholide D

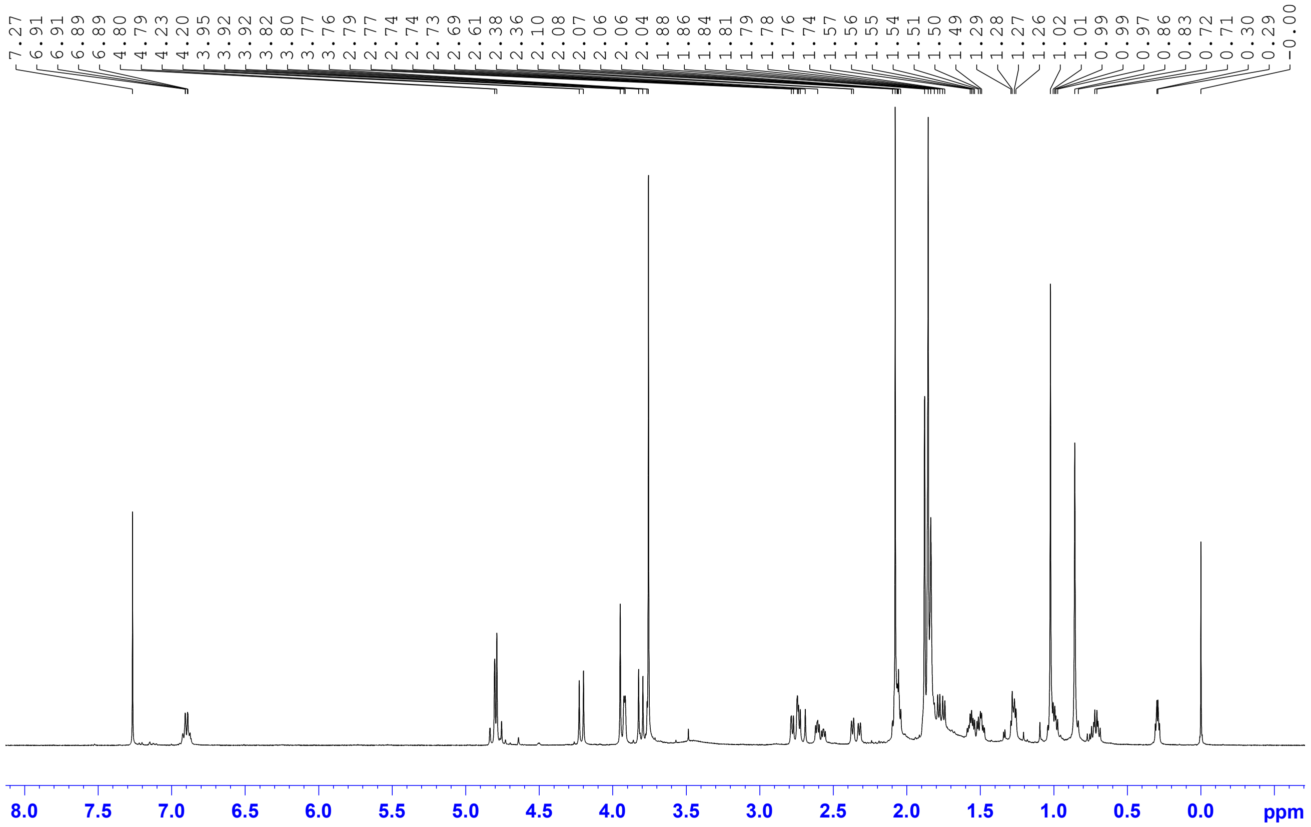


### Figure S2. $^{13}C$ NMR spectrum of chloraholide D

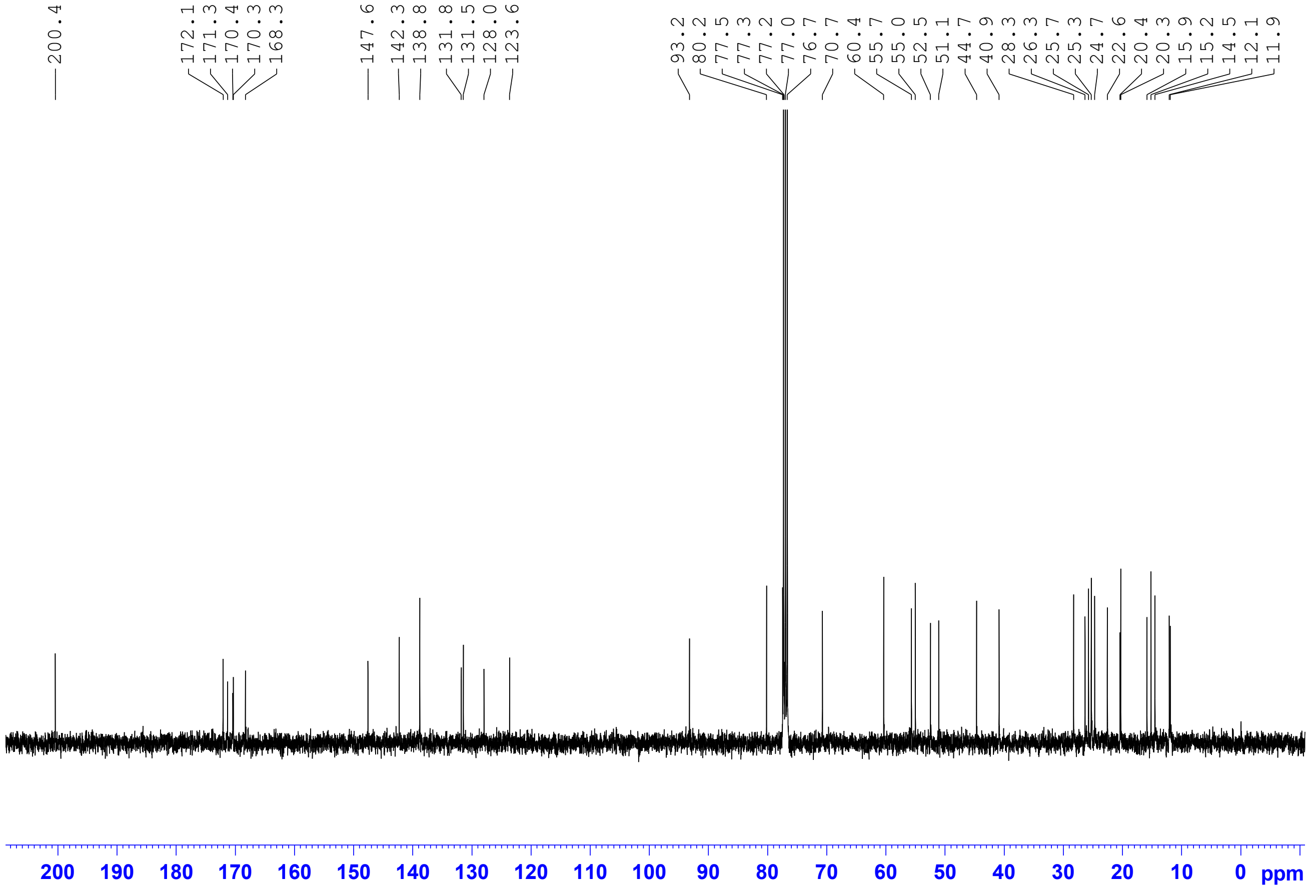

**Figure S3. ¹H NMR spectrum of sarcandrolide A**

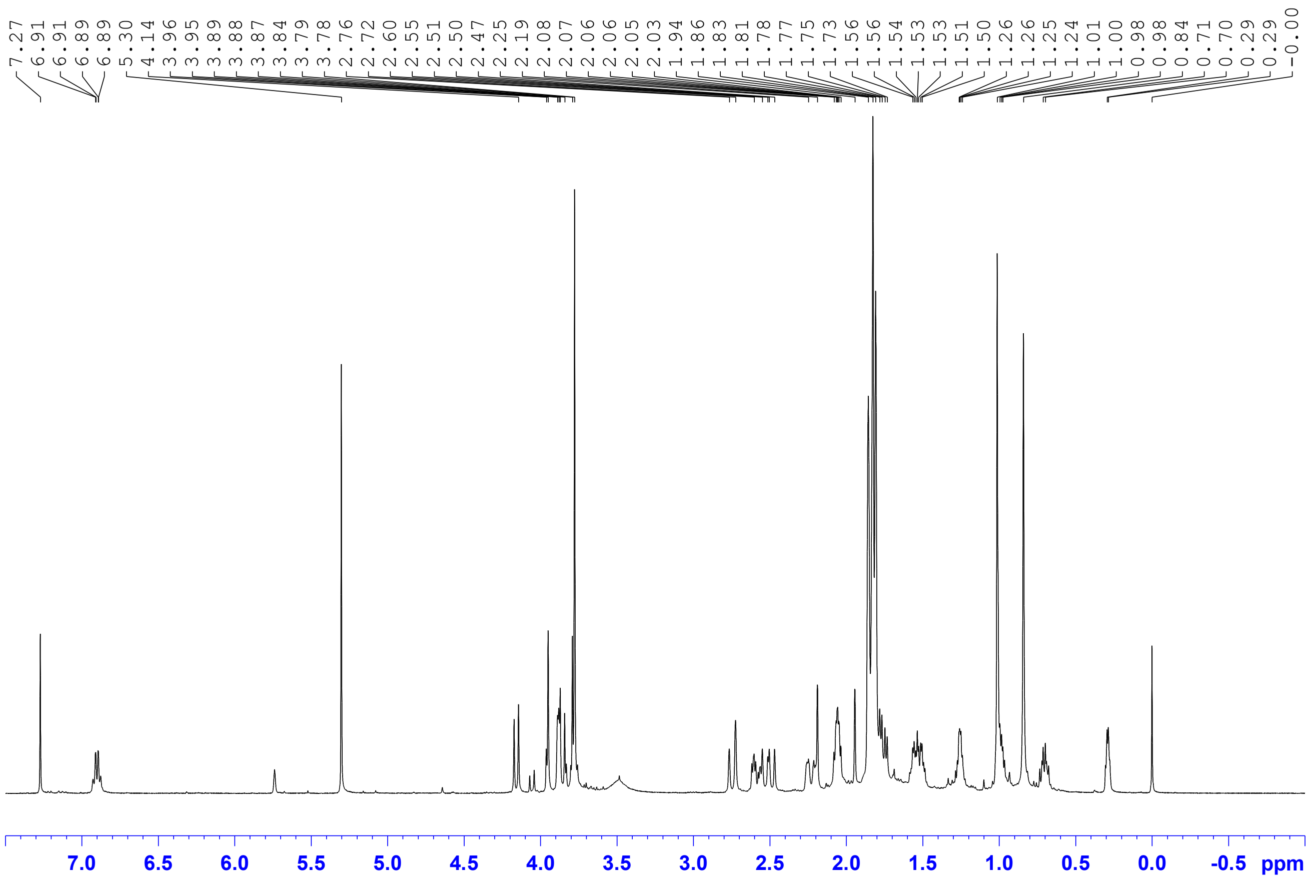

**Figure S4. ¹³C NMR spectrum of sarcandrolide A**

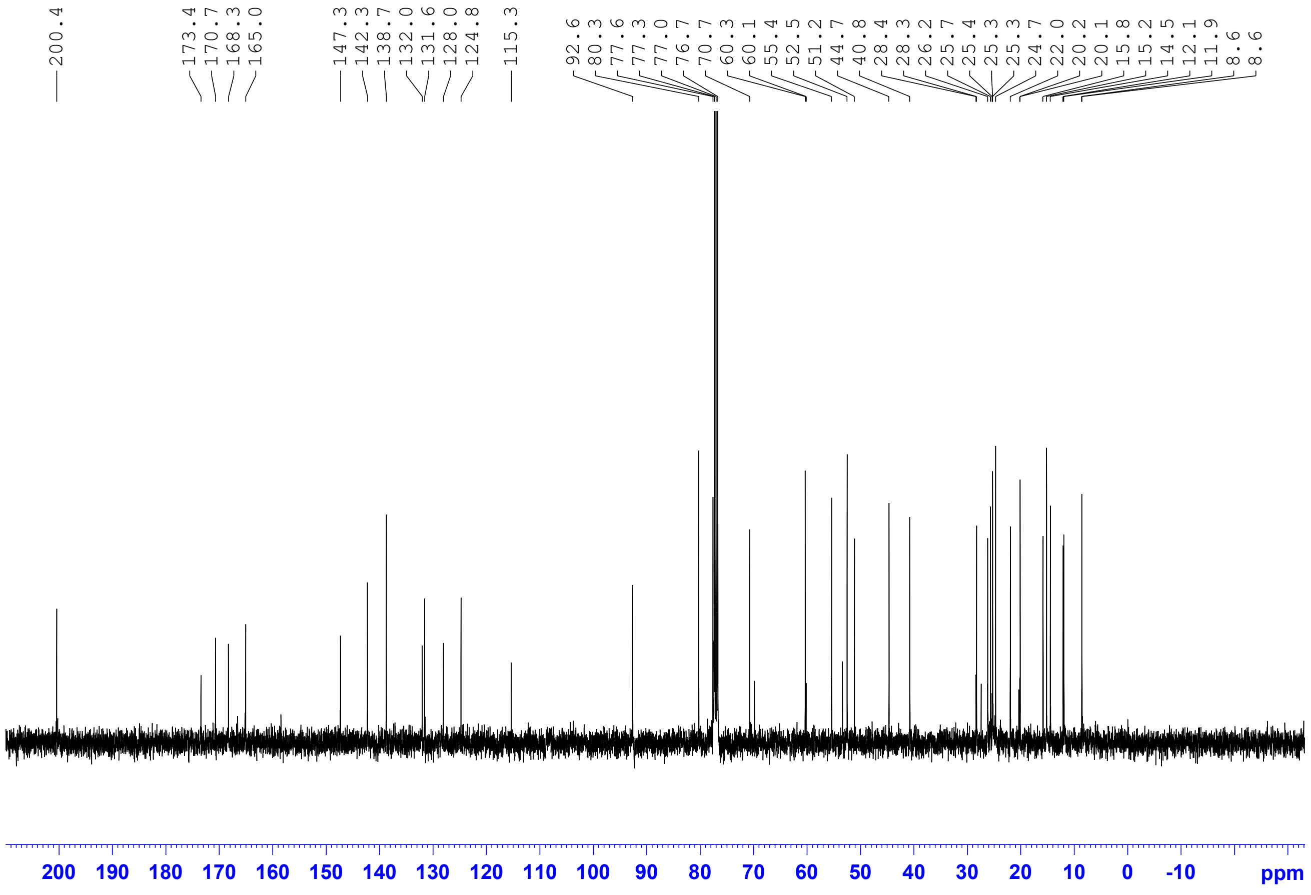

**Figure S5. $^{1}$H NMR spectrum of shizukaol E**

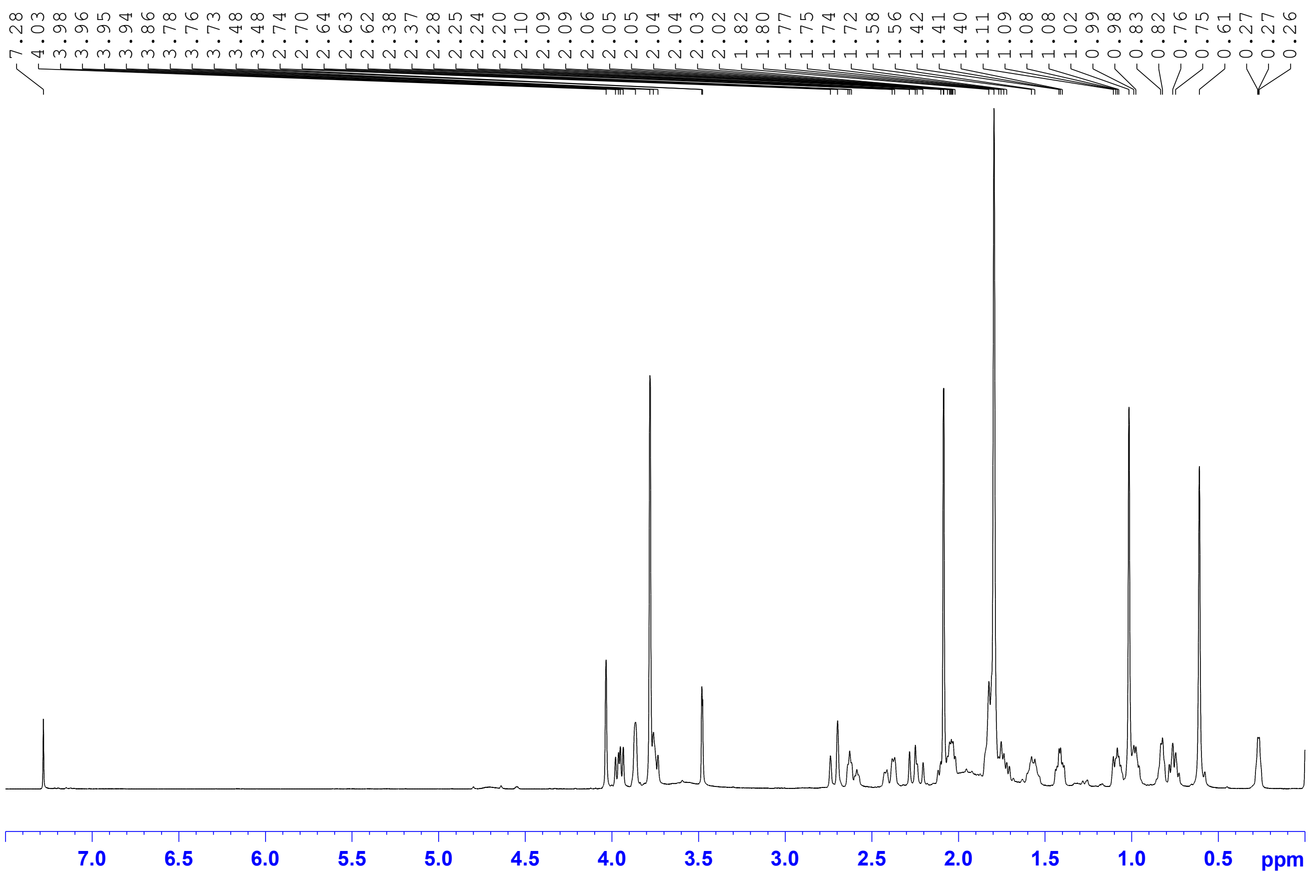


**Figure S6. $^{13}$C NMR spectrum of shizukaol E**

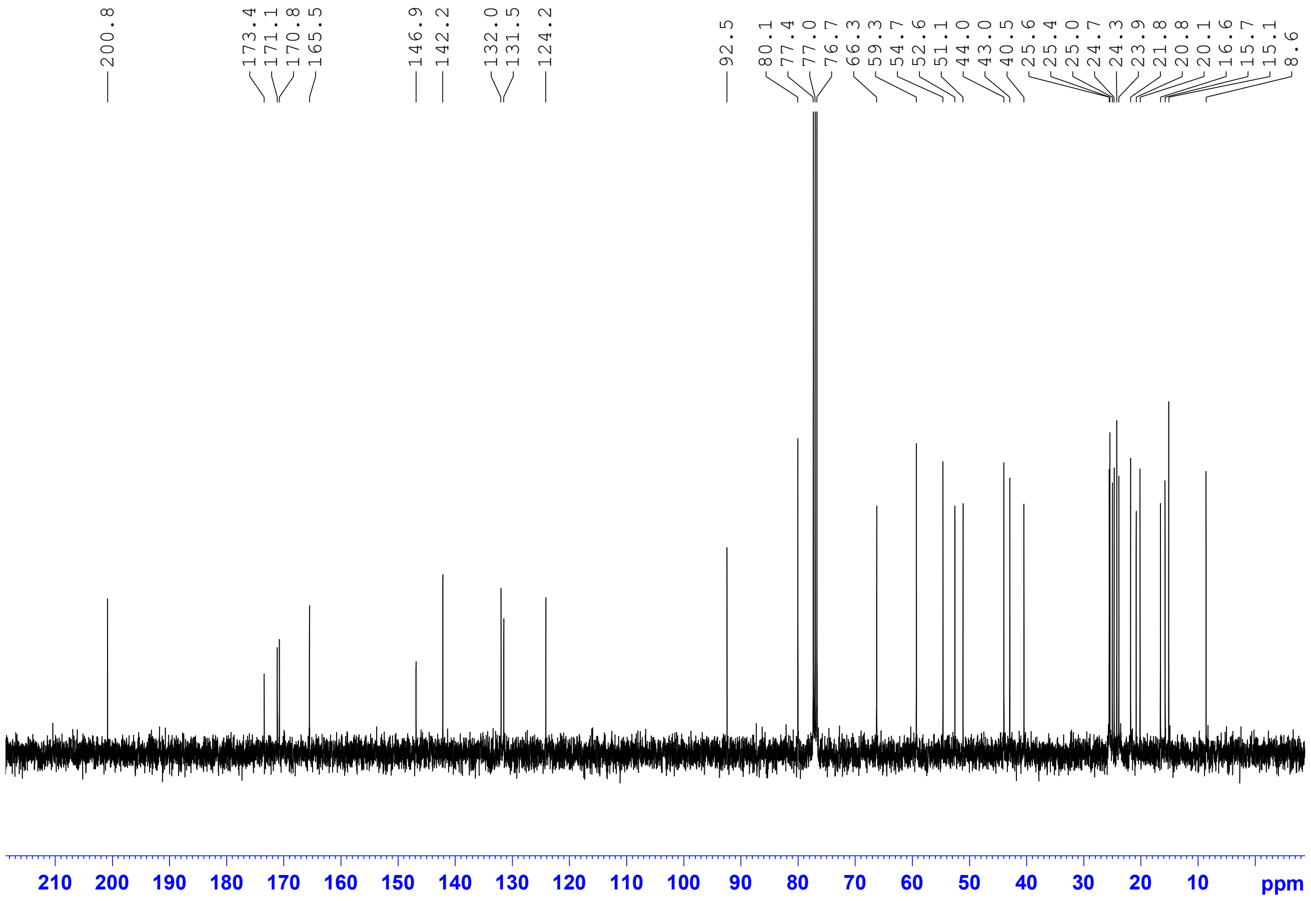

Supplement: Supplementary file 1 [file molecules-28-07070-s001.zip › molecules-2647177-supplementary.pdf]
